# Supplementary material for: DMRforPairs: identifying Differentially Methylated Regions between unique samples using array based methylation profiles
Source: BMC Bioinformatics. 2014 May 15;15:141. doi: 10.1186/1471-2105-15-141 (PMC4046028; doi:10.1186/1471-2105-15-141)
Supplement: Additional file 2 — DMRforPairs output for the comparison of A431-MCF7 and NA17018-NA17105. Please start from the HTML files in each folder. Available via the BMC Bioinformatics website. [file 1471-2105-15-141-S2.zip › 1394847754114233_MOESM2_ESM/A431_MCF7/figures/7503.pdf]

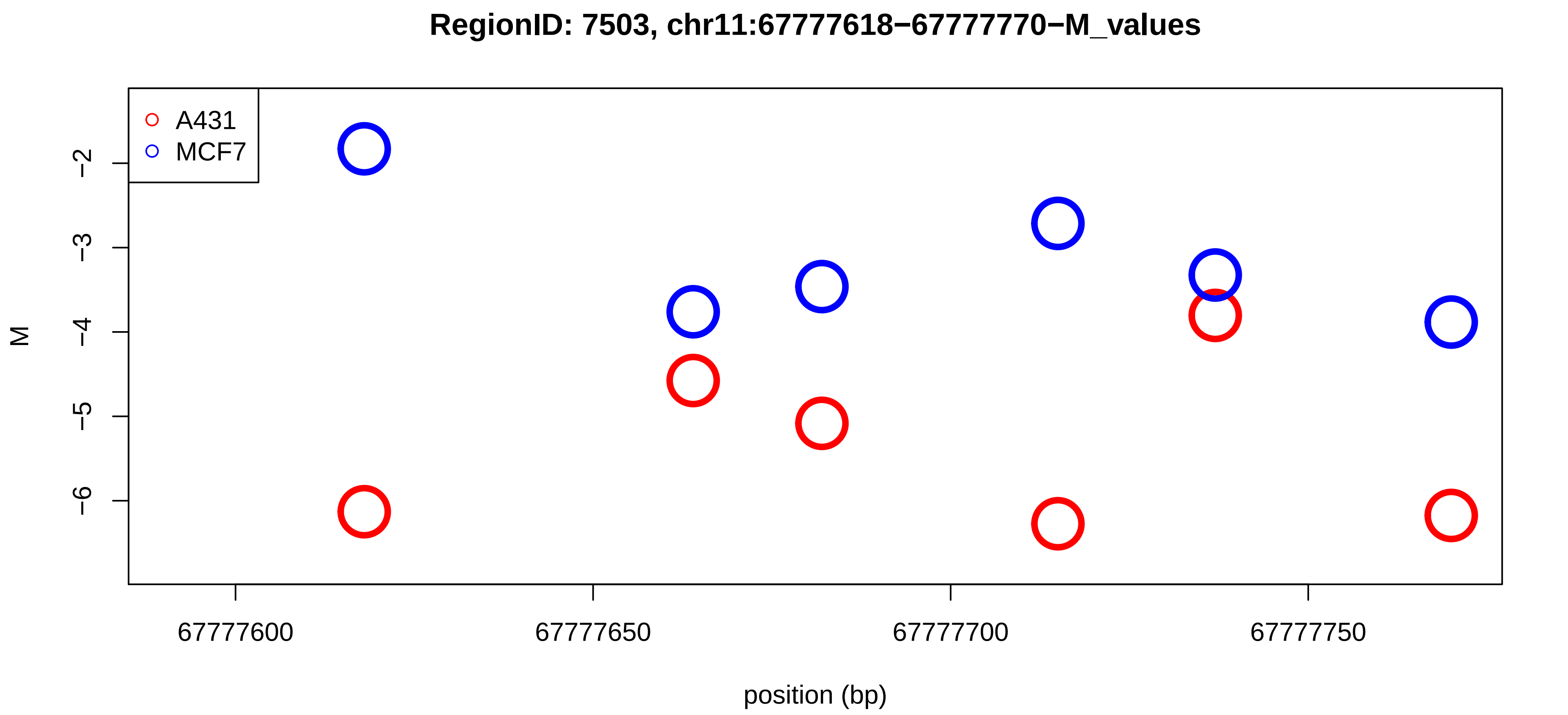
RegionID: 7503, chr11:67777618−67777770−M_values
A431
MCF7
M
−2
−3
−4
−5
−6
67777600
67777650
67777700
67777750
position (bp)

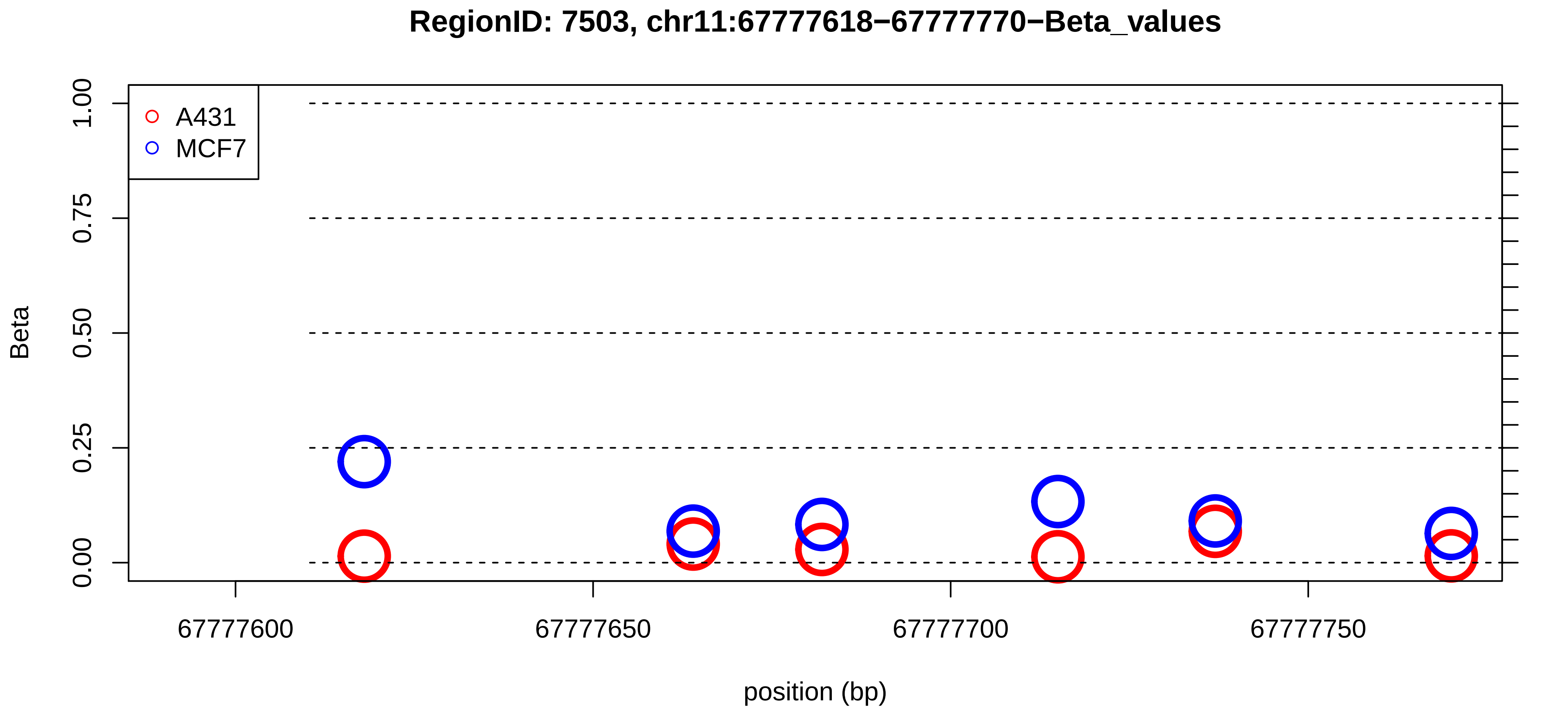
RegionID: 7503, chr11:67777618−67777770−Beta_values
A431
MCF7
Beta
0.00
0.25
0.50
0.75
1.00
67777600
67777650
67777700
67777750
position (bp)

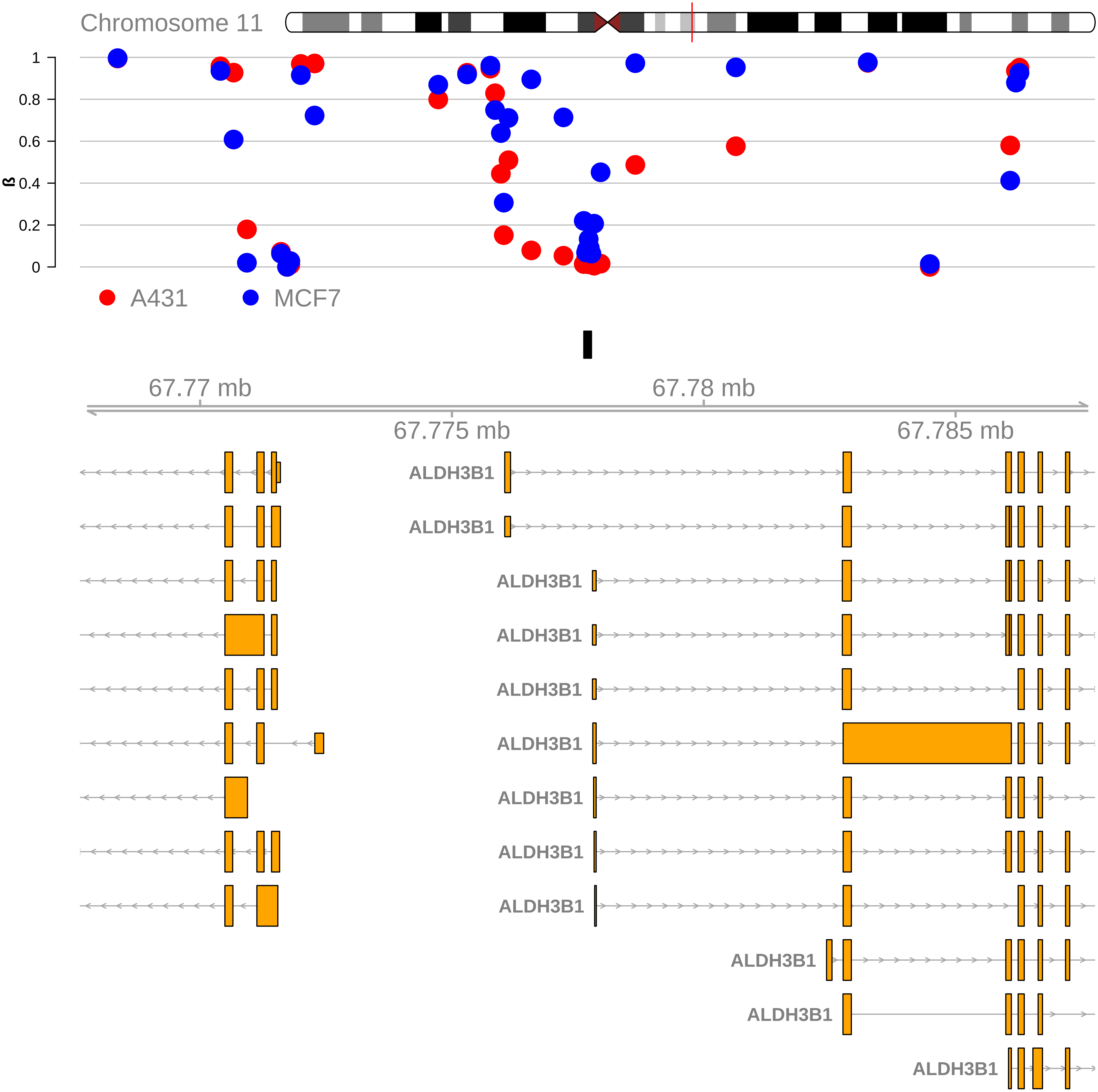

Chromosome 11
1
0.8
0.6
ß 0.4
0.2
0
A431
MCF7
67.77 mb
67.78 mb
67.775 mb
67.785 mb
ALDH3B1
ALDH3B1
ALDH3B1
ALDH3B1
ALDH3B1
ALDH3B1
ALDH3B1
ALDH3B1
ALDH3B1
ALDH3B1
ALDH3B1
ALDH3B1
